# Human Neutrophils Respond to Complement Activation and Inhibition in Microfluidic Devices

**DOI:** 10.3389/fimmu.2021.777932

**Published:** 2021-11-24

**Authors:** Sinan Muldur, Douangsone D. Vadysirisack, Sharan Ragunathan, Yalan Tang, Alonso Ricardo, Camil Elie Sayegh, Daniel Irimia

**Affiliations:** ^1^ BioMEMS Resource Center, Department of Surgery, Massachusetts General Hospital, Harvard Medical School, Boston, MA, United States; ^2^ Shriners Burns Hospital, Boston, MA, United States; ^3^ Ra Pharmaceuticals, Inc., Cambridge, MA, United States

**Keywords:** complement, neutrophil, microfluidic “lab-on-a-chip, phagocytosis, infection, eculizumab, avacopan, RA101295

## Abstract

Complement activation is key to anti-microbial defenses by directly acting on microbes and indirectly by triggering cellular immune responses. Complement activation may also contribute to the pathogenesis of numerous inflammatory and immunological diseases. Consequently, intense research focuses on developing therapeutics that block pathology-causing complement activation while preserving anti-microbial complement activities. However, the pace of research is slowed down significantly by the limitations of current tools for evaluating complement-targeting therapeutics. Moreover, the effects of potential therapeutic agents on innate immune cells, like neutrophils, are not fully understood. Here, we employ microfluidic assays and measure chemotaxis, phagocytosis, and swarming changes in human neutrophils *ex vivo* in response to various complement-targeting agents. We show that whereas complement factor 5 (C5) cleavage inhibitor eculizumab blocks all neutrophil anti-microbial functions, newer compounds like the C5 cleavage inhibitor RA101295 and C5a receptor antagonist avacopan inhibit chemotaxis and swarming while preserving neutrophil phagocytosis. These results highlight the utility of microfluidic neutrophil assays in evaluating potential complement-targeting therapeutics.

## Introduction

Complement activation is key to fast anti-microbial responses, both directly, by damaging the microbes (by the assembly of the membrane attack complex - MAC), and indirectly, by stimulating cellular immune defenses. At the same time, excessive and deregulated activation of complement has been implicated in the pathogenesis of numerous inflammatory and immunological diseases (1, 2), including sepsis (3–5), acute respiratory distress syndrome (6), rheumatoid arthritis (7), glomerulonephritis (8), multiple sclerosis (9), ischemia-reperfusion injury (10), asthma (11), and antineutrophil cytoplasmic antibody-associated-(ANCA) vasculitis (12). Consequently, several anti-inflammatory strategies have emerged based on drugs designed to target upstream, central, and terminal components of the complement cascade, each having advantages and potential risks (13–15). Eculizumab (ECU, Soliris, Alexion Pharmaceuticals, Boston, MA) was the first approved therapy specifically inhibiting the complement pathway. ECU, as well as the newer version with prolonged half-life, Ravulizumab, inhibits the enzymatic cleavage of C5, thus blocking the formation of C5a, C5b, and MAC assembly, which inserts into lipid bilayers forming lytic pores (16). ECU is now an FDA-approved treatment of paroxysmal nocturnal hemoglobinuria, atypical hemolytic uremic syndrome, generalized myasthenia gravis, and Neuromyelitis Optica Spectrum Disorder (17). More recently, a modified macrocyclic peptide inhibitor of C5 cleavage, Zilucoplan (Ra Pharmaceuticals, Cambridge, MA, now part of UCB Pharma), has shown positive phase II data in generalized myasthenia gravis (18). Zilucoplan binds to a distinct site compared to ECU, resulting in the inhibition of both C5 cleavage and C5b binding to C6 and the inhibition of rare C5 variants, such as R885H, identified in non-responder patients to ECU (19). Finally, both C5a antagonists and C5a receptor 1 (C5aR1 or CD88) antagonists have recently entered clinical trials. For example, the C5aR1 antagonist avacopan (AVA, ChemoCentryx, Mountain View, CA) has shown therapeutic efficacy in ANCA-associated vasculitis in a phase III clinical study, supporting the utility of C5aR1 antagonists in human disease (20).

Rare cases of human C5 deficiencies have been associated with recurrent infections, particularly meningitis and extragenital gonorrhea by Neisseria species (21, 22). Similar infections have been associated with the use of complement-targeting therapies in patients, suggesting that these drugs also interfere with the immune responses against pathogens. For example, patients receiving ECU have a higher risk for meningococcal infections (23, 24). Consequently, targeted inhibition strategies, which block the pathology-causing complement activation solely while preserving a level of anti-microbial complement activity and immune surveillance, are the goals of new complement therapeutics (13, 14). Towards this aim, a deeper understanding of how complement affects innate immune responses is essential.

Fundamental for research and drug discovery, *ex vivo* cell-based assays are essential tools for studying cellular immune responses. While *in vivo* imaging allows direct visualization of cellular interactions in the most relevant context, *ex vivo* assays enable the analyses of cellular function in precisely controlled and reproducible conditions. Among the cell-based assays, recent approaches combining microfluidic devices with time-lapse microscopy have enabled the study of neutrophil functions at a single-cell resolution, using minute volumes of blood from healthy subjects or patients. Examples of studies enabled by the new tools include the probing of neutrophil chemotaxis signatures in response to various chemoattractants (25–27), neutrophil neutralization of various pathogens (28), and the cooperation between neutrophils during swarming against clusters of microbes (29, 30). The benefits of microfluidic tools for probing the activity of neutrophils from patients have been demonstrated in sepsis (31, 32), diabetes (33), trauma and burn injuries (34, 35), chronic granulomatous disease (29), organ transplant (27, 36) and Alzheimer’s disease (37). Bridging the clinical and basic research spaces, microfluidic assays for quantifying neutrophil activities represent attractive diagnostic, monitoring, and drug screening tools.

Here, we employ microfluidic tools to characterize human neutrophil responses *ex vivo* to complement activation and investigate the impact of C5 cleavage inhibitors and C5aR1 antagonists. We show that the C5-cleavage inhibitor ECU inhibits neutrophil chemotaxis, phagocytosis, and swarming functions. In contrast, we show that the C5aR1 antagonist AVA and RA101295, a zilucoplan analog (Ra Pharmaceuticals) that has been shown to reduce organ damage and reduced mortality in a sepsis study in primates (38) only inhibit chemotaxis and swarming functions while preserving phagocytosis. Thus, the preservation of neutrophil phagocytosis efficiency, crucial for the control and clearance of pathogenic bacteria, significantly distinguishes AVA and RA101295 from ECU. This observation suggests that these new compounds may preserve some of the anti-microbial protection in C5-associated diseases better than ECU. If this observation is corroborated by clinical evidence, it may help devise new strategies to reduce one of the common side effects of complement-targeting therapeutics.

## Materials and Methods

### Microfluidic Device Designs and Fabrication

Microfluidic devices for testing neutrophil chemotaxis and phagocytosis were designed using AutoCAD (San Rafael, CA). Chrome masks for photolithography were manufactured by high resolution printing (Front Range Imaging, Boulder, CO). Silicon wafers were spin-coated with two layers of negative photoresist (SU-8, Microchem, Newton, MA) using standard microfabrication techniques at the BioMEMS Core at Massachusetts General Hospital (Boston, MA). Two photoresist layers with a thickness of 2 μm for the first layer (chemotaxis channels) and 75 μm for the second layer (cell-loading chambers) were employed for the tapered-chemotaxis device. Three photoresist layers of 5 μm thickness for the first layer (migration channel), 50 μm for the second layer (inner reservoir), and 200 µm for the third layer (outer chamber), were employed for the phagocytosis devices. The wafers were then patterned by sequential ultraviolet light exposure through two or three photolithography masks. The photoresist was processed per the manufacturer’s instructions. The patterned wafers were later used as molds for PDMS (Polydimethylsiloxane, Fisher Scientific, Fair Lawn, NJ). After curing, individual devices were diced using a scalpel. Inlet holes were punched using a 1.2-mm punch (Harris Uni-core, GE Healthcare). Devices were then irreversibly bonded to glass-bottom well plates, as previously described (26–28).

For the swarming assays, a microarray printing platform (Picospotter PolyPico Galway, Ireland) was used to print a solution of 0.1% poly-l-lysine (Sigma-Aldrich) and ZETAG targets with 100 µm diameter. Eight by eight arrays were spotted in a sixteen-well format on ultra-clean glass slides (Fisher Scientific) then dried at 50°C for 2 hours on a heated block. A 16-well ProPlate (Grace Bio-labs) was attached to glass slides. A 50 µL suspension of Texas red-labeled zymosan A *S. cerevisiae* Bioparticles (ThermoFisher Scientific) was added to each well and incubated with rocking for 5-10 minutes. Wells were then thoroughly washed out with PBS to remove unbound targets from the glass surface (29, 30).

### Neutrophil and Plasma Isolation

Fresh samples of peripheral blood from healthy volunteers were purchased (Research Blood Components LLC, Allston, MA). Blood was collected in 10 mL heparin or EDTA tubes (BD Vacutainer, BD, Franklin Lakes, NJ) and utilized within 6 hours after the blood draw. Neutrophils were isolated from EDTA tubes using the EasySep Direct Human Neutrophil isolation kit per the manufacturer’s protocol (Stemcell Technologies, Vancouver, Canada). Isolated neutrophils were stained with Hoechst 33342 (32 µM, ThermoFisher Scientific) and re-suspended in Hanks′ Balanced Salt solution (HBSS, Millipore Sigma) with 0.5% BSA (Millipore Sigma) at a concentration of 12 × 10^6^ cells ml^-1^. Human plasma was collected from heparin tubes by centrifuging at 4°C for 30 minutes at 3000 rpm.

### Neutrophil Chemotaxis Assay

The microfluidic device for probing neutrophil chemotaxis in conditions of progressive mechanical confinement consists of an array of tapered channels, with cross-section progressing from 20 µm^2^ to 6 µm^2^ (10 µm to 3 µm width, 500 µm in length, and 2 µm uniform height) (27). The neutrophil chemotaxis channels are connected to one shared cell-loading channel and an array of chemoattractant chambers. The chemoattractant chambers were filled either with fMLP (N-formyl-methionyl-leucyl-phenylalanine, 100 nM, Millipore Sigma), recombinant human C5a protein (ab61918, 0.1 - 10 µM, Abcam), or C3a (3677-C3-025, 0.1 - 10 µM, R&D Systems) diluted in HBSS with 0.5% BSA during the initial priming step. A chemoattractant gradient is established along the migration channels when the neutrophils suspended in HBSS with 0.5% BSA are introduced in the device. All the concentrations reported for the chemotaxis experiments are those in the end chamber. The concentrations in the cell-loading channels, when chemotaxis is initiated, are roughly one tenth of the concentrations in the end chamber. The local concentrations in the cell-loading channels have been validated in previous work and are consistent with the optimal concentrations that trigger human neutrophil chemotaxis (27).

For endogenous complement activation, plasma collected from heparin blood was stimulated with different concentrations of cobra venom factor (CVF, 10 to 100 µg/ml, Millipore Sigma) for 30 min and introduced into the device. To inhibit C5 cleavage, plasma was pretreated with ECU (3 µM in PBS) or RA101295 (3 µM in 0.3% DMSO, Fisher Scientific) and allowed to equilibrate for at least 30 minutes. The 3 µM concentration for inhibitors is representative of therapeutically relevant concentrations achieved in the blood of patients treated with ECU (17) and zilucoplan (18). To account for potential DMSO effects, 0.3% final concentration was added to no-inhibitor controls. Finally, isolated neutrophils in HBSS and 0.5% BSA were pretreated (or not) with AVA (Avacopan, MedChemExpress, Monmouth Junction, NJ, USA; product number HY-17627, 0.05-10 µM in 0.3% DMSO) for at least half an hour before being pipetted into the cell-loading chamber of the device. Neutrophil chemotaxis was monitored for 5 hours using time-lapse imaging (one image every 5 minutes).

We probed the effect of complement in the presence of serum on neutrophil chemotaxis by suspending them in Iscove’s Modified Dulbecco’s Medium (IMDM, Sigma I3390) with 20% Fetal Bovine Serum (FBS, ThermoFisher Scientific). The chemoattractant chambers were also filled with recombinant human C5a protein (ab61918, 0.1 and 1 µM, Abcam) diluted in IMDM with 20% FBS during the priming step. A chemoattractant gradient is established along the migration channels when the neutrophils are introduced in the device.

### Neutrophil Phagocytosis Assay

The devices for measuring neutrophil phagocytosis consist of dozens of target chambers (200 μm diameter × 50 μm height) arrayed inside a larger outer channel (50 μm height). The target chambers are connected to the outer channel by narrow migration channels (125 μm long × 10 μm height × 10 μm width) (28). The target chambers were loaded with *Staphylococcus aureus* bioparticles (*S. aureus*, ThermoFisher) during priming of the devices. The bioparticles were labeled with Alexa-488 and pHrodo (Life Technologies, Carlsbad, CA) and suspended in plasma with either ECU (3 µM) or RA101295 (3 µM). Human C5-depleted serum (234405, Sigma) was used as a negative control. Isolated neutrophils in HBSS with 0.5% BSA were then loaded in the outer channel after pre-treatment with either AVA (250 nM), ECU (3 µM), or RA101295 (3 µM) for at least half an hour. Additionally, neutrophils were labeled with Hoechst and pHrodo dyes (ThermoFisher). Chemotaxis and phagocytosis were monitored for 5 hours using time-lapse imaging (one image every 5 minutes).

### Neutrophil Swarming Assay

Arrays of 100 µm diameter clusters of Texas red-labeled zymosan *(S. cerevisiae* derived bioparticles, ThermoFisher) in microwells were submerged under plasma pretreated with ECU (3 µM) or RA101295 (3 µM) [25]. Human C5-depleted serum (234405, Sigma) was used as the negative control. Isolated neutrophils were suspended in HBSS with 0.5% BSA and pretreated with BLT1/2 antagonists (U-75302 and LY255283, 40 µM, Cayman chemical), and CXCR1/2 (MAB331-100, 2 µg/ml, R&D Systems) and AVA (250 nM) for at least half an hour before being added to each well (500,000 cells/well). Neutrophil swarming around the zymosan clusters was monitored for 8 hours by time-lapse imaging (one image every 2 minutes).

### Image Acquisition and Analysis

Time-lapse images of neutrophil chemotaxis, phagocytosis, and swarming were monitored over time using brightfield and fluorescence (DAPI, FITC, CY3) at 10 X magnification with a fully automated Nikon TiE inverted wide-field microscope with a bio chamber heated to 37°C with 5% CO2. Image analysis for the neutrophil chemotactic assay was performed with ImageJ/FIJI (National Institutes of Health) software to track and analyze neutrophils trajectories. Percentages of neutrophil chemotaxis were calculated based on the total number of cells migrating into the channels over the total number of cells present in the cell loading chamber at time zero for each field of view. We analyzed 252 tapered channels (21 tapered channels per field of view and 12 fields of views per condition) per experiment and condition tested. Persistent migration indicates neutrophils that migrated through the channels without changing directions. Arrest describes neutrophils that are trapped in the channels. Oscillation indicates neutrophils that change migration direction more than two times. Retrotaxis describes neutrophils that migrated back to the cell-loading channel. Image analysis for neutrophil phagocytosis assay was performed with ImageJ/FIJI software. Percent phagocytosis of *S.aureus* bioparticles was calculated by dividing the total surface area of free over the phagocytosed particles present at the end of the experiment in each field of view. We calculated an average of the percent phagocytosis and absolute neutrophil recruitment obtained for each of the 9 central chambers per experiment and condition. Image analysis for the neutrophil swarming assay was performed with ImageJ/FIJI software. Swarm area analysis was performed manually by outlining the swarms. Intensity profiles were generated over time by defining regions of interest. We analyzed 4 swarms per experiment and per condition.

### Flow Cytometry for Internalized Particles

We employed traditional flow cytometry techniques to assess neutrophil internalization of *S. aureus* bioparticles labeled with Alexa-488 and pHrodo. Neutrophils were pre-incubated with AVA, whereas human sera (Complement Technology, Inc., Tyler, TX, USA; product number NHS) were pre-incubated with C5 inhibitors (ECU, ECU F(ab)_2_ divalent fragment following papain digestion, or RA101295) for 10 minutes at room temperature. Pretreated neutrophils and human sera were then incubated together with *S. aureus* bioparticles for 45 minutes at 37°C. Samples were placed on ice and treated with EDTA (5 mM final concentration) to immediately inhibit further phagocytosis and complement activation. Nonspecific cell surface fluorescence signal was quenched with 0.08% trypan blue. Neutrophils were then analyzed for internalized bioparticles, identified by double fluorescence (Alexa-488 and low-pH activated pHrodo), using MACSQuant Analyzer 10 flow cytometer.

### C5a Measurements

We employed a solid-phase enzyme-linked immunosorbent assay (ELISA) based on the sandwich principle for measuring human C5a concentrations, according to the manufacturer’s protocol (Quidel Corporation, San Diego, California, USA; product number A021). The kit is accompanied with a TMB substrate solution. Supernatant from flow cytometry internalized particle experiments were diluted 300-fold to a final serum concentration of 0.125%. The absorbance at 450 nm is measured with a spectrophotometer. The human C5a concentration of samples was calculated from the absorption and the standard curve.

### Generation of the Eculizumab F(ab’)2 Fragment

Eculizumab was processed using the PierceTM F(ab’)2 Preparation Kit (ThermoFisher, Waltham, MA, USA, Catalog #44988) and in accordance with the manufacturer’s instruction. Briefly, 1 mg of eculizumab was desalted with the Zeba Spin Desalting column. Desalted eculizumab solution (0.2 mL) was mixed with 0.3 mL of digestion buffer and incubated with pepsin resin at 37°C for 4 hours. The digestion product was passed through the Nab Protein A Plus column at a final volume of 2 mL followed by overnight dialysis with PBS (pH 7.4) at 4°C using a 20K MWKO Slide-A-LyzerTM Dialysis Cassette (ThermoFisher, Waltham, MA, USA Catalog #66033).

### Statistical Analysis

We used Prism (GraphPad Software version 8.3) for statistical analyses and graphs. Values are presented in the results and in the figures as mean ± SD (standard deviation). For swarming experiments, values are presented as mean ± SEM (standard error of the mean). Data were first tested for normality using the Shapiro-Wilk test normality test. Normally distributed data comprised of more than two groups was analyzed using parametric One-Way ANOVA with Dunnett’s post-hoc test. Non-normally distributed data were analyzed with a non-parametric Kruskal-Wallis test, followed by Dunn’s post-hoc test, where appropriate. Data consisting of two groups were analyzed with Student’s two-tailed t-test. Differences between means were considered significant at p<0.05. Probability values are indicated on figures by asterisks, as follows: *p < 0.05; **p < 0.01; ***p < 0.001; ****p < 0.0001. The absence of significant differences is marked as ns (not significant).

## Results

**Figure 1:**
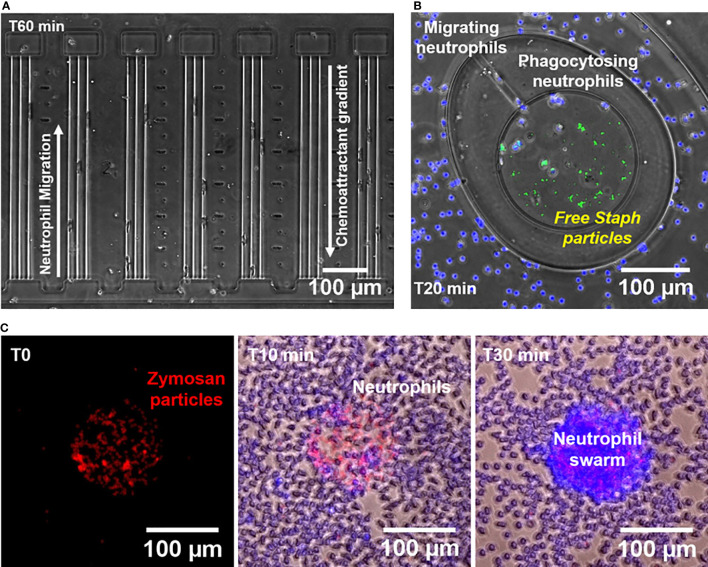
Microfluidic devices for measuring neutrophil chemotaxis, phagocytosis, and swarming behaviors. **(A)** Microscopic image of a segment of the chemotaxis microfluidic device (x10 Brightfield, Nikon TiE) showing neutrophil chemotaxis towards C5a and C3a during increasing mechanical restriction. Each chemoattractant chamber is connected to the cell loading channel through 3 tapered channels. **(B)** Microscopic image of a segment of the phagocytosis microfluidic device (x10 Brightfield/Fluorescent, Nikon TiE) showing neutrophils (blue nuclei) from the outer chamber migrating towards the central reservoir through a migration channel to phagocytose *S. aureus* particles (green) in plasma at T_20_ minutes. **(C)** Microscopic images obtained at three different time points (T_0_, T_10_, and T_30_ minutes) showing the formation of a neutrophil swarm (blue nuclei) over a cluster of Texas red-labeled zymosan A S. cerevisiae bioparticles (Red).

We investigated the ability of human neutrophils to migrate directionally in response to gradients of C5a and C3a (**Figure 1A**), heat-killed-*S. aureus* particles, (**Figure 1B**), and *Saccharomyces cerevisiae* cell-wall derived bioparticles (zymosan particles, **Figure 1C**). We employed assays that have been recently developed and the methods to analyze the data have been validated.

### Neutrophil Chemotaxis Towards C5a and C3a Through Small Channels

**Figure 2:**
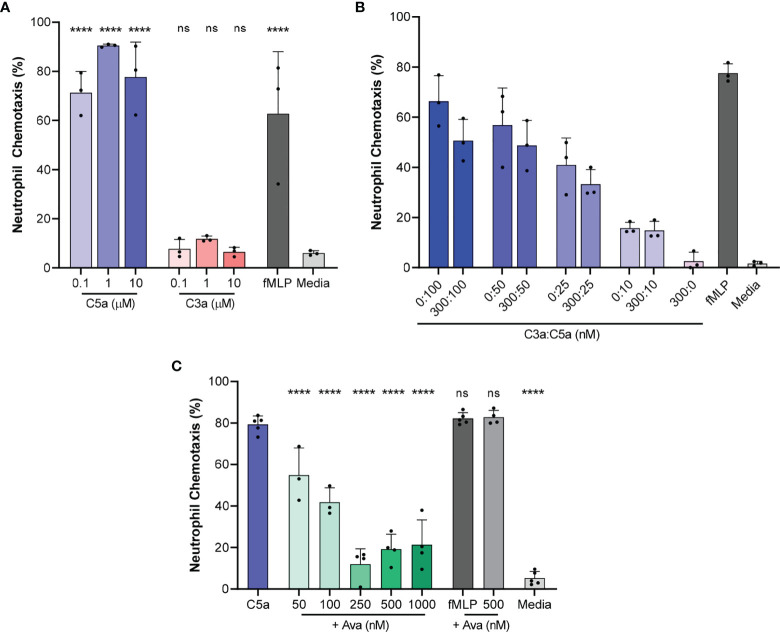
Chemotaxis of isolated neutrophils towards C5a and C3a and the effect of AVA on chemotaxis. **(A)** The percentage of neutrophils migrating directionally towards increasing concentrations of C5a and C3a compared to media alone (HBSS with 0.5%BSA, N=3, ****p < 0.0001; One-way ANOVA with Dunnett’s test) **(B)** C3a inhibits neutrophil chemotaxis towards C5a, C5a effect is dependent on concentration, and there is no interaction effect between C3 and C5a. (N=3 donors, p < 0.005, Repeated measures, two way ANOVA, with Sidak’s correction for multiple comparisons). **(C)** Percentage of neutrophils migrating directionally towards C5a (100 nM) after treatment of neutrophils with C5aR1 antagonist AVA (N=3, 4 or 5, each dot on the plot represents one experiment, ****p < 0.01 for comparisons to C5a control; ns represents not statistical significant. One way ANOVA with Dunnett’s multiple comparison test with single pooled variance).

We determined that 71.3 ± 8.7%, 90.5 ± 0.6% and 77.7 ± 14.3% of the human neutrophils migrated directionally towards 0.1, 1, and 10 µM C5a concentrations in the chambers, respectively (N=3 experimental repeats, N=1161, 1232, 1430 neutrophils counted for each of the three conditions, **Figure 2A**). In positive control experiments, 62.8 ± 25.2% of the neutrophils responded to chemoattractant 100 nM fMLP (N=3, N=1861, **Figure 2A**). In negative control experiments, 5.9 ± 1% neutrophils migrated towards media alone (HBSS with 0.5% BSA, N=3, N=1067, **Figure 2A**). The fraction of neutrophils migrating in the chamber was 21.8 ± 14.1%, 40.7 ± 20.8%, and 64.5 ± 11.4%, at C5a concentrations of 1, 10, and 50 nM in the chamber, respectively (N=3, N=847, 584, 639 respectively, **Figure S1**). At these concentrations, the fractions of moving cells were statistically comparable to those in negative control media (p>0.05, N=3, **Figure S1**).

In contrast to the response to C5a, only 7.7 ± 3.9%, 11.8 ± 1.2% and 6.5 ± 1.9% responded to 0.1, 1, and 10 µM C3a, respectively (N=3, N=1424, 1262, 1494 respectively, **Figure 2A**). Multiple-comparison tests revealed that C5a and fMLP induced chemotaxis in a significantly larger percentage of neutrophils compared to the media alone (p < 0.0001, One-way ANOVA with Dunnett’s test). The differences between C3a-induced chemotaxis and media alone were not significant, suggesting that C3a is a less potent human neutrophil chemoattractant at concentrations between 100 nM and 10 µM **(**N=3, N=1424, 1494 respectively **Figure 2A** **)**.

In addition, the fraction of neutrophils responding to C5a at 100 nM was significantly diminished when C5a was mixed in IMDM with 20% FBS compared to HBSS with 0.5% BSA (p < 0.001; unpaired two-tailed t-test, N=3, **Figure S2**). The reduction in the effectiveness of C5a in the presence of serum has been previously studied in detail (39) and may be due to the presence of factors in the serum, such as carboxypeptidases, which could remove the C-terminal arginine and lysine residues from the C5a (desargination), reducing the effect on neutrophils several fold (40). To avoid the potential confounding effect of serum when measuring the effect of complement-targeting therapeutics, we supplemented the culture media with 0.5% BSA in all our subsequent experiments.

Furthermore, we studied the effect on neutrophil chemotaxis of combinations of C5a and C3a using one C3a concentration (300 nM) and various C5a concentrations (10, 25, 50, and 100 nM). For every C5a concentration tested, we observed consistently fewer neutrophils migrating towards C5a and C3a in combination compared to C5a alone, at different C5a concentrations. For example, 50.6 ± 8.5% of neutrophils migrated towards a combination of 300 nM C3a and 100 nM C5a **(**N = 3, N = 958, 1160, respectively, **Figure 2B**) compared to 66.4 ± 10.2% neutrophils migrating directionally in response to 100 nM C5a. A repeated-measures two way ANOVA revealed that the effect of C3a is statistically significant (N = 3, p < 0.005, repeated measures, two way ANOVA, with Sidak’s correction for multiple comparisons). Our analysis confirmed that the effect of C5a is dose dependent and showed that the effect of C3a is consistent at the various C5a concentration tested.

### AVA Blocks C5a-Induced Neutrophil Chemotaxis

We determined the effect of AVA on neutrophil chemotaxis towards C5a at 100 nM concentration. This concentration of C5a was determined in previous experiments to stimulate the chemotaxis of the largest fraction of neutrophils. Following pretreatment with increasing concentrations of AVA 50, 100, 250, 500 nM and 1 µM, the fraction of neutrophils migrating directionally decreased from 79.3 ± 4.1% (C5a 100 nM, N=5, N=1493) to 54.9 ± 13.1%, 41.8 ± 6.9%, 11.9 ± 7.4%, 19.2 ± 7.2% and 21.3 ± 12%, respectively (N=3, N=774; N=3, N=974; N=4, N=1482; N=4, N=1480; N=4, N=1602; **Figure 2C**). AVA at a concentration of 250 nM appeared to be most effective at blocking chemotaxis towards C5a (100 nM, p<0.0001 when compared to C5a, One way ANOVA with Dunnett’s multiple comparison test with single pooled variance). A separate comparison of the neutrophil chemotaxis % values in the presence of various AVA concentrations to those at 250 nM shows that the chemotaxis at the 500 and 1000 AVA are not significantly different than the 250 nM. Moreover, the chemotaxis levels are not statistically different from the media control. In additional control experiments, we determined the effect of AVA on the neutrophil chemotaxis towards fMLP (100 nM), demonstrating the specificity of AVA to the neutrophil responses to C5a.

### Neutrophil Migration Patterns Induced by C5a

**Figure 3:**
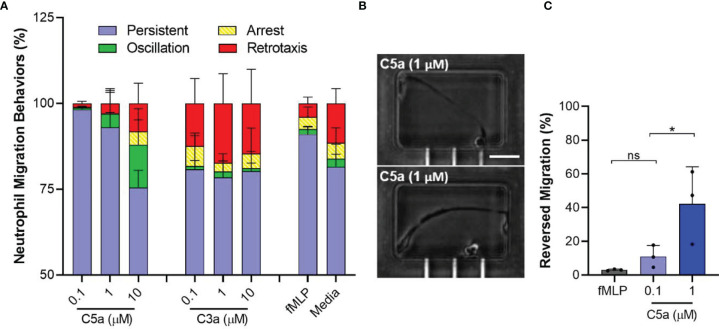
Atypical membrane elasticity of neutrophils and migration patterns when exposed to high C5a dose. **(A)** Percentage of neutrophil migration patterns during chemotaxis towards C5a, C3a, and fMLP. (N=3). **(B)** Microscopic images of elongated neutrophils when exposed to C5a 1µM inside the end chambers. Scale = 100 µm. **(C)** Percentages of unique reversed migration phenotype of neutrophils observed when exposed to high C5a concentration. (N=3, *p < 0.05; ns represents not statistical significant. One-way ANOVA with Dunnett’s test).

We analyzed the migration patterns of C5a-induced neutrophil migration in tapered channels, including persistent chemotaxis, arrest, oscillation, and retrotaxis (**Figure 3**). We found that the arrest, oscillation, and retrotaxis occurred more frequently during chemotaxis towards 1 and 10 µM C5a concentration compared to 100 nM C5a and 100 nM fMLP (N=3, **Figure 3A**). We found that neutrophils exposed to C5a at 1 µM and above concentrations undergo dramatic changes in shape, acquiring extremely elongated morphologies (**Figure 3B**). For example, the percentages of reversed migration were higher at 1 µM C5a compared to 100 nM C5a and 100 nM fMLP (42.3 ± 21.9%, 11.0 ± 6.6%, and 3.0 ± 0.5% respectively, N=3, N=642, 755, 687 respectively; p < 0.05, One-way ANOVA with Dunnett’s test, **Figure 3C**). We also observed that neutrophils exposed to 1 µM C5a inside the end chamber leave the chamber and undergo retrotaxis through the tapered channel, back to the initial cell loading chamber. These observations suggest that higher doses of C5a may desensitize the neutrophils and interfere with their ability to migrate directionally. The small number of neutrophils that migrated in response to C3a displayed a higher frequency of retrotaxis, comparable to neutrophils in the negative control condition.

### Neutrophil Chemotaxis Towards Endogenously Generated Complement Is Reduced in the Presence of AVA, ECU, and RA101295

**Figure 4:**
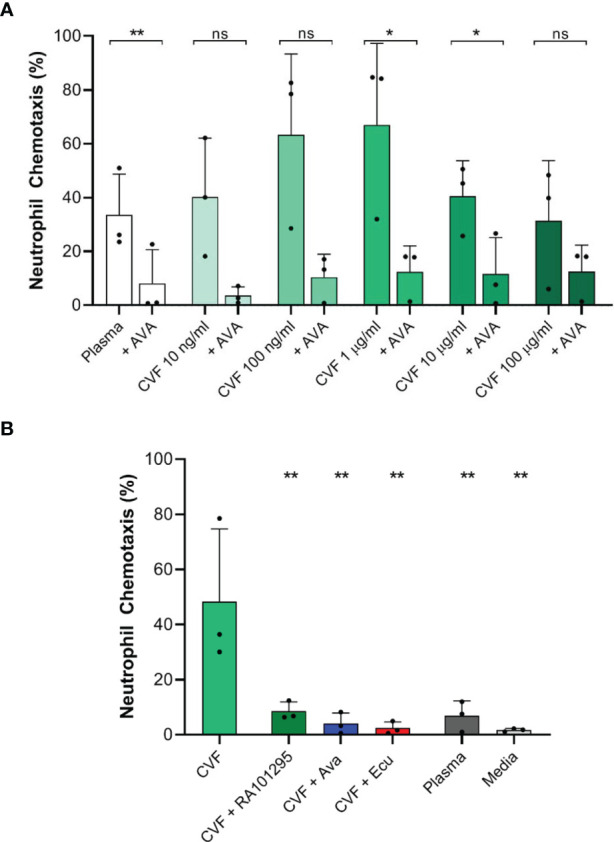
Effect of C5 complement inhibitors on neutrophil chemotaxis towards endogenous complement activation with CVF. **(A)** Effect of AVA (250 nM) on endogenous complement activation by increasing CVF doses (N=3 donors, *p < 0.05, **p < 0.01; paired two-tailed *t*-test). **(B)** Effect of C5 inhibition with ECU (3 µM) and RA101295 (3 µM) on endogenous complement activation by CVF (N=3, **p < 0.01; ns represents not statistical significant. One-way ANOVA with Dunnett’s test).

We studied neutrophil chemotaxis towards endogenously activated complement. In response to human plasma stimulated with 0.01, 0.1, 1, 10 and 100 µg/ml CVF we observed 40.1 ± 22%, 63.2 ± 30%, 67 ± 30.3%, 40.5 ± 13.1% and 31.4 ± 22.4% neutrophils migrating directionally towards the activated complement, respectively, (**Figure 4A**, N=3, N=1676, 1419, 1373, 1378, 1168, 1170 respectively). These values were higher compared to 33.5 ± 15.2% migrating in response to plasma alone. When we pretreated neutrophils with 250 nM AVA the percentages of neutrophils migrating towards CVF activated plasma were reduced 2 to 5-fold to 3.6 ± 3.2%, 10.4 ± 8.5%, 12.4 ± 9.6%, 11.7 ± 13.4% and 12.5 ± 9.7%, respectively (**Figure 4A**, N=3, N=1925, 1226, 1568, 1202, 931, 2461 respectively). The differences were statistically significant for CVF at 1, 10 µg/ml, and plasma alone (p<0.05, p<0.05, p<0.01, respectively; paired two-tailed t-test). These results also suggest the presence of low C5a levels in plasma controls, likely generated during the contact of plasma with the glass and PDMS surfaces of the devices. Moreover, these results are consistent with the lower chemotaxis in the presence of media compared to plasma (**Figure 4B**).

We also studied neutrophil chemotaxis when plasma was pretreated with C5-cleavage inhibitor ECU (3 µM) and RA101295 (3 µM) prior to CVF stimulation (1 µg/ml). CVF stimulation of plasma induced significant chemotaxis of 48.3 ± 26.3% neutrophils compared to plasma alone with 6.8 ± 5.5% (**Figure 4B**, N=3, N=1128 and 1288 respectively). When plasma was pretreated with ECU, RA101295 or neutrophils were pretreated with AVA, we observed a significant decrease of neutrophil chemotaxis to respectively 2.40 ± 2.3%, 8.55 ± 3.4%, and 4.04 ± 3.87% compared to CVF treated plasma (**Figure 4B**, N=3, N=1171, 1139 and 1131 respectively). The differences were statistically significant for all C5 inhibitors (p < 0.01, respectively; One-way ANOVA with Dunnett’s test). Overall, the use of ECU, AVA, and RA101295 reduced the number of neutrophils migrating towards CVF by approximately 75%.

### ECU, AVA, and RA101295 Reduce Neutrophil Swarming

**Figure 5:**
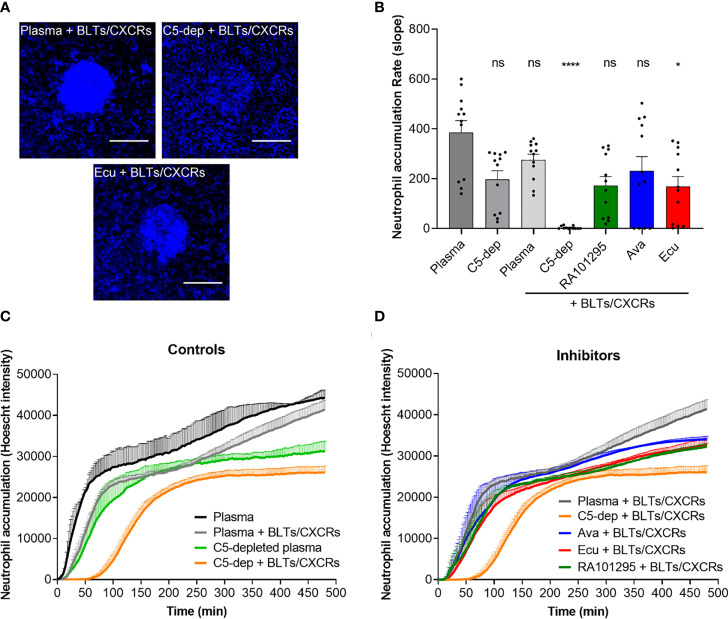
Evaluation of C5-inhibitors guiding neutrophil migration during swarming towards zymosan particle clusters. **(A)** Microscopic fluorescent images of neutrophil recruitment and swarm formation after one hour in plasma, C5-depleted, and ECU treated plasma with neutrophils pretreated with LTB4 (BLTs) and IL8 (CXCRs) inhibitors. Neutrophils nuclei are labeled in blue (Hoechst 33342). The scale bar is 100 µm. **(B)** Effect of C5 inhibition of plasma with ECU, AVA, and RA10129 on swarming kinetics. Only the effect of ECU reached statistical significance relative to untreated plasma. Each dot represents the slope value of each zymosan-particle cluster during the first hour (*p < 0.05, ****p < 0.0001; Kruskal-Wallis with Dunn’s test). **(C)** Neutrophil recruitment and accumulation kinetics over time in plasma and C5-depleted plasma in the absence or presence of LTB4 (BLTs) and IL8 (CXCRs) inhibitors neutrophil pre-treatment (N=3). **(D)** Neutrophil recruitment and accumulation kinetics over time in plasma pretreated with ECU, RA101295, or AVA in the presence of LTB4 (BLTs) and IL8 (CXCRs) inhibitors neutrophil pre-treatment (N=3). ns represents not statistical significant.

We measured the neutrophil swarming towards 100 µm diameter clusters of zymosan bioparticles (**Figure 1C**) in plasma pretreated with C5-cleavage inhibitors (ECU and RA101295). In plasma controls, swarming responses of neutrophils are usually very fast events, starting around 10 minutes to 30 minutes after their addition to the assay. While LTB4 and IL8 are the major signals driving the swarming process, blocking the relevant BLT1 and CXCR1 receptors, can outline the role of C5a during swarming. Qualitatively, in the presence of BLT1 and CXCR1 antagonists, we observed complete swarms at one hour in plasma, absent swarming in C5-depleted plasma, and incomplete swarms with ECU treated plasma (**Figure 5A**). The rate of neutrophil accumulation around zymosan-particle clusters decreased during the first hour in the presence of BLT1 and CXCR1 antagonists. The inhibitory effects of RA101295, AVA and ECU were comparable, and only ECU reached statistical significance (p < 0.05; Kruskal-Wallis with Dunn’s test **Figure 5B**). Swarming slowed down around 60 minutes and reached a plateau after 90 minutes **(**N=3 experiment, N=12 swarms observed, **Figure 5C** **)**. The kinetics of swarming is delayed by 1 hour, and the plateau is reached after 4 hours in the presence of BLT1 and CXCR1 antagonists and C5-depleted plasma compared to plasma controls **(**N=3, N=12, **Figure 5C** **)**. Complement inhibitors ECU and RA101295 did not significantly change the dynamics of swarming in the presence of BLT1 and CXCR1 antagonists **(**N=3, N=12, **Figure 5D** **)**. Similarly, neutrophils pretreated with AVA displayed no significant changes during swarming in the presence of BLT1 and CXCR1 antagonists **(**N=3, N=12, **Figure 5D** **)**. We noted a larger variability in the size of the swarms during the first two hours for AVA-treated neutrophils compared to the other two complement inhibitors. The effect of C5 depleted serum on swarming, distinct from that of the inhibitors, suggests that C5 depletion has impacted other pathways in addition to C5 activation (**Figure 5D**).

### AVA and RA101295 but Not ECU Preserve Neutrophils Phagocytosis of *S. aureus* Bioparticles

**Figure 6:**
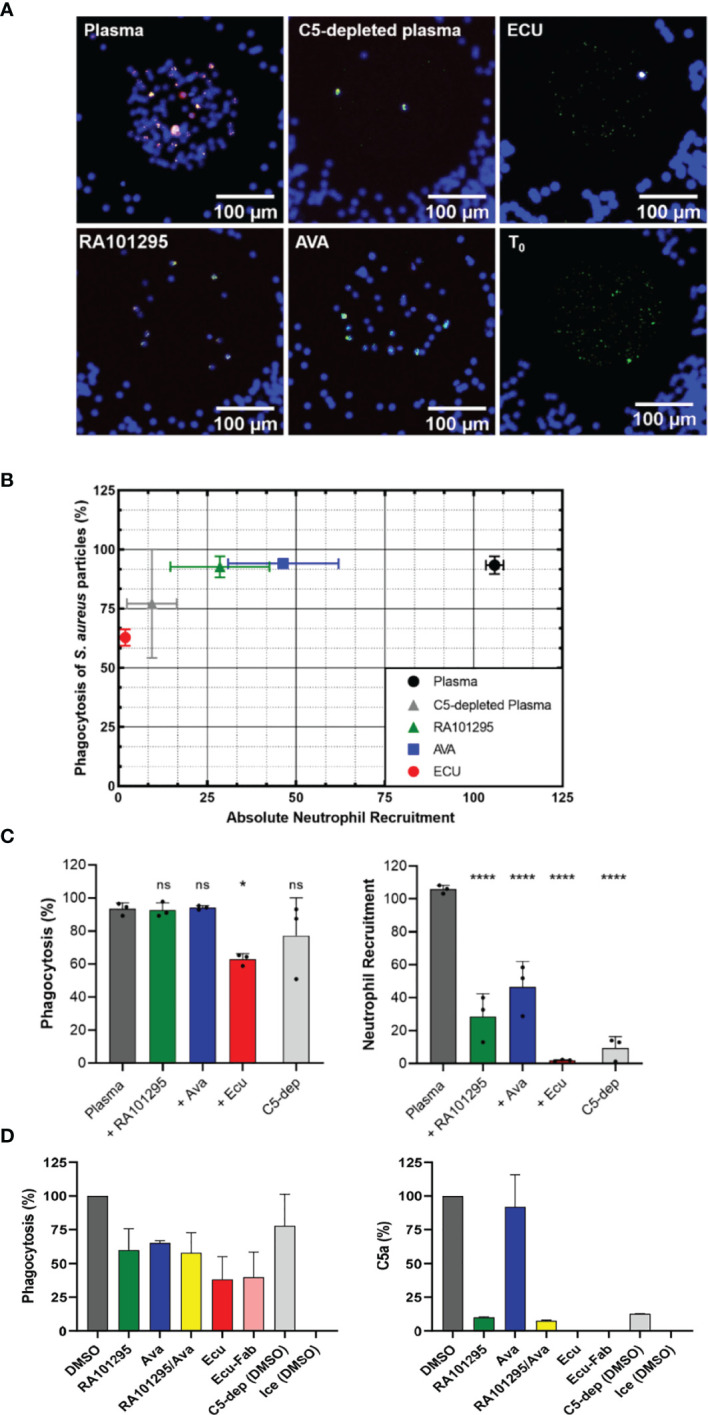
Effect of complement inhibitors on neutrophil phagocytosis behavior of *S. aureus* -particles. **(A)** Microscopic fluorescent images of the central reservoir of the device at the end of an experiment, showing neutrophils nuclei labeled in blue (Hoechst 33342), *S. aureus* particles labeled in green (FITC), and phagosome acidification in red (pHrodo) once phagocytosed by neutrophils. T_0_ shows the initial number of *S. aureus* particles present in the central reservoir before chemotaxis and phagocytosis events. **(B)** End-point results of phagocytosis percentage *vs.* recruitment of neutrophils in plasma pretreated with ECU, RA101295, and AVA pretreated neutrophils. **(C)** Effect of C5 inhibition with ECU (3 µM), AVA (250 nM), and RA101295 (3 µM) on percentages of *S. aureus* particles phagocytosis and neutrophil recruitment numbers (N=3) (*p < 0.05; ****p < 0.0001; One-way ANOVA with Dunnett’s test). Only ECU-treated plasma affects phagocytosis of neutrophils significantly when compared to untreated plasma. Plasma pretreated with all three inhibitors decreases neutrophil recruitment significantly. **(D)** Neutrophil phagocytosis using flow cytometry for internalized particles. RA101295 (3 µM) reduced particle phagocytosis less than AVA and ECU. C5a concentration in sera shows that RA101295 exhibits residual C5 cleavage (C5a generation), unlike ECU or ECU-Fab. The combination of RA101295 and AVA did not further inhibit phagocytosis as compared to either agent alone (N=2). ns represents not statistical significant.

We studied neutrophil phagocytosis of *S. aureus* bioparticles (**Figure 1B**) in plasma pretreated with C5-cleavage inhibitors (ECU and RA101295, respectively) and C5aR1 antagonist AVA. Microscopic fluorescent images of the central reservoir of the device at the end of experiments showed that the accumulation of neutrophils was reduced, and the fraction of *S. aureus* particles phagocytosed was altered by different amounts by ECU, RA101295, and AVA (**Figure 6A**). Neutrophil-*S. aureus* particle interactions in complete plasma resulted in robust chemotaxis (105.9 ± 2.4 neutrophils entering the chambers) and efficient phagocytosis of *S. aureus* particles (93.4 ± 3.8%) (N=3 experiments, N=27 chambers, **Figures 6B, C**). In C5-depleted plasma, neutrophils showed weak chemotaxis (9.4 ± 7.0 cells/chamber) and a moderate decrease in *S. aureus* particles phagocytosis (77.2 ± 22.9%, **Figures 6B, C**). Pretreating plasma with ECU significantly inhibited both chemotaxis (1.9 ± 0.5 cells/chamber) and phagocytosis (62.8 ± 3.5%) when compared to plasma (p < 0.0001 and p < 0.05, respectively; One-way ANOVA with Dunnett’s test, **Figure 6C**). The recruitment and phagocytosis were lower than the C5-depleted plasma. The effect of ECU on neutrophil recruitment and *S. aureus* phagocytosis was the same when ECU was inside the chambers only (with plasma) or when present everywhere in the device (with neutrophils, **Figure S3**). Pre-treatment of neutrophils with AVA reduced neutrophil chemotaxis by half (46.4 ± 15.6 cells/chamber) when compared to plasma (p < 0.0001, **Figures 6B, C**). However, AVA had minimal effect on phagocytosis (94.1 ± 1.27%). We observed the same effect when AVA was added only to the chambers or everywhere inside the device (**Figure S3**). Pre-treatment of neutrophils with RA101295 reduced neutrophil chemotaxis to roughly one quarter (28.6 ± 14 cells/chamber) when compared to plasma (p < 0.0001). Interestingly, RA101295, similarly to AVA, had no apparent effect on phagocytosis (92.7 ± 4.5%, **Figures 6B, C**). We observed a slight increase in neutrophil recruitment when RA101295 was present everywhere in the device (**Figure S3**). The phagocytosis ability of neutrophils in the presence of RA101295 in the chamber and everywhere in the device was unchanged and comparable to that of untreated controls (**Figure S3**). The temporal kinetics of neutrophil recruitment towards *S. aureus* particles and the phagocytosis of these particles was diminished in the presence of ECU and C5-depleted plasma compared to RA101295 and AVA (**Figure S4**). The evaluation of neutrophil phagocytosis using flow cytometry for internalized particles revealed changes in the presence of the various inhibitors that were, in general, consistent with the results of the microfluidic assay (**Figure 6D**). Treatment of RA101295 or AVA alone or in combination reduced neutrophil phagocytosis to a lesser extent than ECU (N=2). The ability of ECU to more potently inhibit phagocytosis was independent of binding to Fc receptors on neutrophils because an ECU-F(ab)2 fragment lacking an Fc domain displayed the same degree of inhibition as full-length ECU. Furthermore, we found that the differential effects of complement inhibitors ECU and RA101295 in neutrophil phagocytosis were aligned with the concentration of C5a generated in sera from the microfluidics and flow cytometry assays (**Figure 6D**). Measurement of C5a from these experiments revealed that RA101295 exhibited residual C5 cleavage as opposed to ECU or ECU-F(ab)2. We also noted the presence of residual C5a in the C5 depleted plasma (**Figure 6D**).

As the percentage of phagocytosis depends on the number of neutrophils recruited in our assay, we compared the phagocytosis efficiency between chambers with identical numbers of neutrophils recruited. For example, by monitoring chambers inside which only four neutrophils were recruited, we found that these neutrophils phagocytosed 92.9% and 56% of *S. aureus* particles after 3 hours in RA101295-treated and ECU-treated plasma, respectively (N=4). This result further shows that ECU limits neutrophil recruitment and significantly impairs neutrophil phagocytosis.

## Discussion

**Figure 7:**
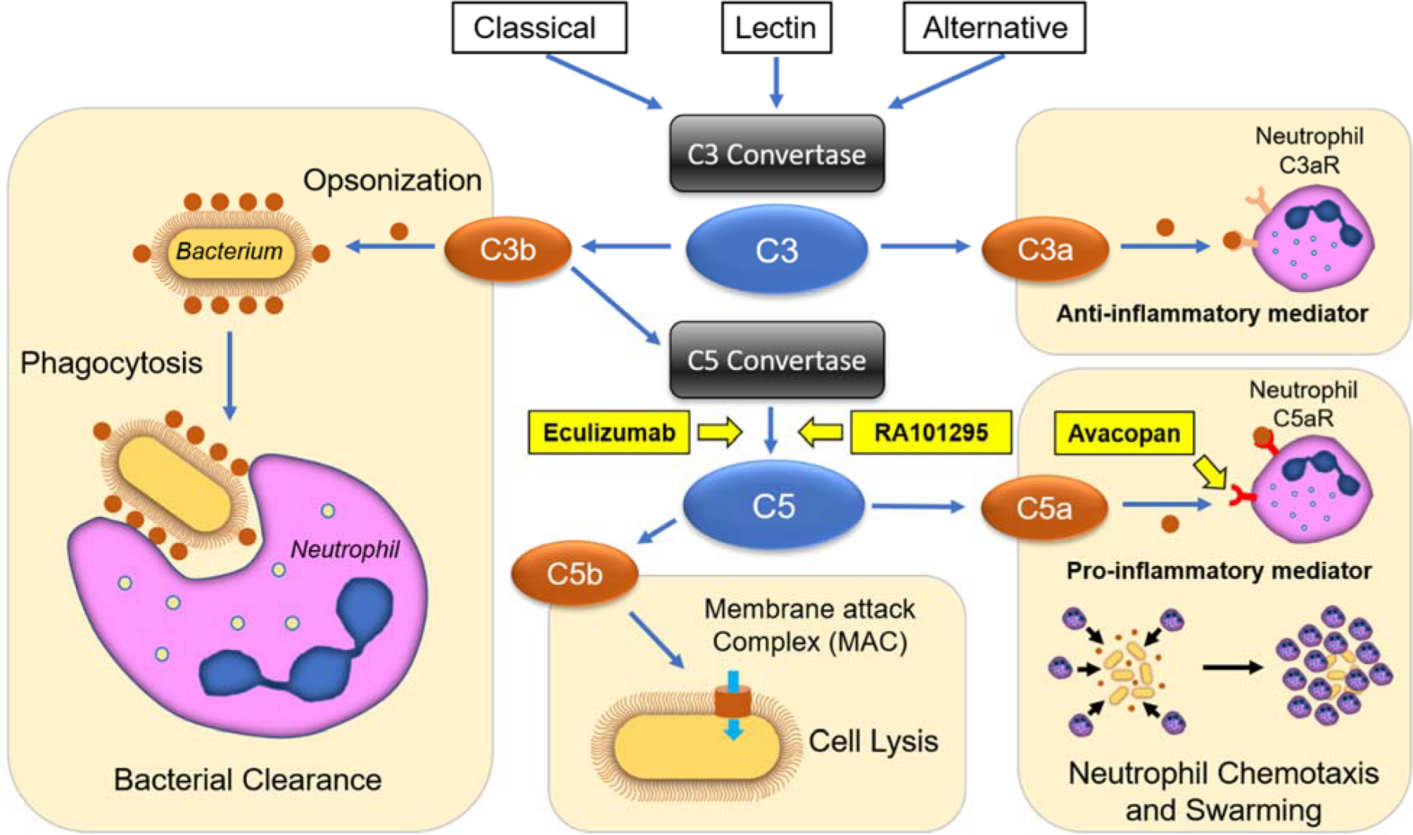
Schematic representation of complement activation pathways, terminal inhibition strategies, and their effects on neutrophil functional behaviors. The classical, lectin, and alternative pathways converge on the cleavage of the central components C3 into C3a and C3b peptides. C3a acts as an anti-inflammatory mediator towards neutrophils, while C3b enhances phagocytosis through opsonization and forms C5 convertases, which cleaves C5 into C5a and C5b. C5a is a potent pro-inflammatory mediator and chemoattractant, while C5b forms the membrane attack complex (MAC), leading to cell and bacteria lysis. ECU and RA101295 are C5-inhibitors, which block the production of C5a and C5b peptides, while AVA blocks C5a mediated chemotaxis and pro-inflammatory effects.

We applied microfluidic assays to measure essential human neutrophil functions: chemotaxis, phagocytosis, and swarming in response to complement factors C5a and C3a, as well as to the complement activation by CVF. We also measured neutrophil function changes in the presence of three complement inhibitors: AVA, ECU, and RA101295, targeting different steps of complement activity (**Figure 7**). Key features of the microfluidic assays for the precision measurements of neutrophil functions include the use of tapered channels to probe neutrophil chemotaxis during increasing mechanical restriction (27), the confinement of phagocytosis targets in microscale arenas that neutrophils could access through one migration channel (28), and the large scale arrays for probing neutrophil swarming against uniform-sized clusters of microbe-like particles (30). Together, these features enabled us to efficiently isolate the effect of the complement factors and inhibitors on each of the three essential neutrophil functions.

We employed tapered microfluidic channels and verified that C5a was a potent neutrophil chemoattractant at 10-100 nM concentrations, comparable to the levels reported in septic patients (3). We verified that the chemotactic activity of C5a is dependent on binding to C5aR1, the target of AVA antagonist. While residual chemotaxis is present at even the highest AVA concentrations, it is likely that other receptors, like the C5aR2 may also be playing a minor role in chemotaxis, as reviewed (41). We found that the highest C5a concentrations increased the oscillatory and reversed migration patterns during chemotaxis. At high concentrations, C5a also induced physical deformation of the neutrophils that took extremely elongated shapes. Although C3a is commonly described as a pro-inflammatory mediator, our study found that it is a less potent neutrophil chemoattractant. The lack of a robust chemotactic response to C3a compared to C5a in our assay may be attributed to differences in expression of cognate receptors on unstimulated neutrophils (42). While our results are consistent with other reports (23, 43), we acknowledge that the role of C3a in stimulating neutrophil chemotaxis remains controversial and may be different than the effect on other granulocytes, e.g. eosinophils (44). Moreover, our research suggests that C3a may be counteracting the effect of C5a, in agreement with some previous reports (45). As expected, endogenous complement activation in the presence of CVF also had potent neutrophil chemoattractant effects. This effect is due to the activation of the CVFBb convertase complex that is stable and cleaves C5 efficiently (46). The neutrophil migration in the presence of CVF at different doses has a bell-curve response that is typical for various chemoattractant molecules, including C5a, acting on G coupled protein receptors (47). The decrease in neutrophil migration at higher than optimal C5a concentrations has been reported before to occur through the downregulation of C5a receptors, either through internalization or receptor shedding on neutrophils (48).

Complement factors, C5a in particular, are responsible for driving pathological inflammatory responses in many diseases (1–11). Consequently, it has been proposed that blocking the C5 activation and blocking C5aR1 signaling would limit the inflammatory damage mediated by neutrophils and other leukocytes. Both strategies would preserve essential upstream components such as C3b-mediated opsonization and phagocytosis while inhibiting C5a-mediated neutrophil chemotaxis. Our experiments confirm that the inhibition of C5 cleavage and C5a-C5aR1 interactions (through the use of RA101295, ECU, and AVA) had a more pronounced effect on chemotaxis than on phagocytosis.

Several studies have revealed that blocking the terminal complement complex (MAC) formation increases the risks for infections (23, 24, 49, 50). For example, C5-inhibitor ECU, which has clinical efficacy in diseases such as paroxysmal nocturnal hemoglobinuria, atypical hemolytic uremic syndrome, generalized myasthenia gravis, and neuromyelitis optica spectrum disorder (17), is also increasing the risk for meningococcal infections (43). Although anti-meningococcal vaccination mitigates the risk for infections, recent studies have reported that vaccinated patients may still contract the meningococcal disease because of impaired bacterial lysis and reduced opsonophagocytic functions in these patients (23, 43). Our data comparing ECU, RA101295, and AVA activities on neutrophil chemotaxis and phagocytosis confirmed the expected deficiency of antimicrobial deficiency of ECU treated neutrophils. By contrast, in the presence of RA101295, neutrophil chemotaxis and phagocytosis were conserved. The priming of neutrophils for phagocytosis may be compensated by the C5a mediated upregulation of CR3 (51, 52). While both RA101295 and ECU can inhibit C5 cleavage, it appears that when faced with strong activating surfaces such as that of bacteria, RA101295 preserves neutrophil phagocytosis better than ECU. RA101295 also preserved neutrophil phagocytic activity in whole blood challenged with E. coli and, in a primate sepsis study, it reduced organ damage and reduced mortality (38). It is worth noting that very small amounts of C5a detected in the presence of RA101295 and in the C5-depleted plasma appear to be sufficient for neutrophil activation. Similar to RA101295, in the presence of AVA, neutrophil chemotaxis and phagocytosis were also conserved. This effect may be explained by the binding of some of C5a produced in the presence of bacteria to C5aR1, despite the competition from AVA. Interestingly, ECU appears to have a more pronounced impact on neutrophil phagocytosis than RA101295. Even though both ECU and RA101295 are C5 cleavage inhibitors, our measurements reported here suggest that the residual C5a in the presence of RA101295 may be sufficient for neutrophil activation. RA101295, similar to its clinical analog zilucoplan, blocks MAC formation through a mechanism of action that disrupts C5b6 complex (18, 53). This finding is consistent with results reporting that RA101295 completely suppressed sC5b9 levels under conditions where residual C5a was detectable using a whole blood assay. (38).

The distinction between the effects of ECU, RA101295, and AVA on human neutrophils was possible using microfluidic assays. These helped resolve the interdependence between chemotaxis and phagocytosis functions during neutrophil activities. In the future, the use of microfluidic assay will help fine-tune the desired effects of new drugs while minimizing the negative effects on neutrophils. Broader use and technical solutions to increase the throughput of microfluidic assays will accelerate the screening of new compounds, while new features will increase the quantitative information available from the *in vitro* assay for each compound.

## Data Availability Statement

The raw data supporting the conclusions of this article will be made available by the authors, without undue reservation.

## Authors Contributions

SM, AR, CES, and DI conceived the study. SM, DV, AR, CES, and DI designed experiments. SM designed and fabricated microfluidic devices. SM, DV, SR, and YT performed experiments. SM, DV, AR, CES, and DI analyzed results. SM, DV, CES, and DI wrote the manuscript. All authors contributed to the article and approved the submitted version.

## Funding

This study was funded in part by Ra Pharmaceuticals (now a part of UCB Pharma), the National Institutes of Health grants (GM092804, EB002503), and Shriners Burns Hospital (#85123 and nano shared facility). The funders were not involved in the study design, collection, analysis, and interpretation of data, the writing of this article or the decision to submit it for publication.

## Conflict of Interest

Authors, DDV, SR, YT, AR and CES were employees of Ra Pharma at the time when this work was performed. DDV, SR, YT, CES and AR were shareholders of Ra Pharma (now part of UCB Pharma).

The remaining authors declare that the research was conducted in the absence of any commercial or financial relationships that could be construed as a potential conflict of interest.

## Publisher’s Note

All claims expressed in this article are solely those of the authors and do not necessarily represent those of their affiliated organizations, or those of the publisher, the editors and the reviewers. Any product that may be evaluated in this article, or claim that may be made by its manufacturer, is not guaranteed or endorsed by the publisher.

## Supplementary Material

Supplementary Figure 1 Percentage of neutrophil migrating directionally in response to C5a gradients is maximal at 0.1 to 10 µM C5a in the end chamber. Neutrophil chemotaxis increased with increasing C5a concentration from 1 nM to 1 µM. 1 nM, 10 nM, 50 nM, 100 nM, 1 µM and 10 µM (N=3, N=3, N=3, N=6, N=3, N=3, respectively) (*p < 0.05, ***p < 0.001; Kruskal-Wallis with Dunn’s test).

Supplementary Figure 2 Effect of serum on C5a-induced neutrophil chemotaxis. Fewer neutrophils respond to C5a at 100 nM in IMDM with 20% FBS compared to serum-free HBSS with 0.5%BSA (***p < 0.001; unpaired two-tailed *t*-test). This effect was not observed for higher C5a doses (1 µM, N=3).

Supplementary Figure 3 Effect of ECU, AVA, and RA101295 on neutrophil recruitment and *S. aureus* particle phagocytosis when the inhibitors are present everywhere in the device versus inside the central reservoirs only. We observed the same effect when ECU and AVA were inside the chambers only (with plasma) or when present everywhere in the device (with neutrophils). RA101295, when present everywhere and with cells, slightly increased neutrophil recruitment. (N=3)

Supplementary Figure 4 Kinetics of neutrophil recruitment and phagocytosis of *S. aureus* -particles in the presence of ECU, AVA, and RA101295. A. Neutrophil recruitment over time. The fastest and strongest neutrophil recruitment was obtained in the positive control (Plasma + *S. aureus*), while the lowest recruitment was obtained in the negative control (C5-depleted + *S. aureus*) and ECU pretreated plasma. RA101295 and AVA reduced neutrophil recruitment as well but to a lesser extent. B. Phagocytosis of *S. aureus* particles by the recruited neutrophils. The fastest phagocytosis of *S. aureus* particles was observed in the positive control (Plasma). Phagocytosis was delayed in the AVA and RA10295 conditions, even though they ultimately reached the same phagocytosis percentages as the positive control. Neutrophils in ECU-pretreated plasma followed a much slower and diminished phagocytosis rate similar to the negative control (C5-depleted) (N = 1).
