# Supplementary material for: Human Neutrophils Respond to Complement Activation and Inhibition in Microfluidic Devices
Source: Front Immunol. 2021 Nov 24;12:777932. doi: 10.3389/fimmu.2021.777932 (PMC8653703; doi:10.3389/fimmu.2021.777932)
Supplement: Supplementary Figure 1 — Percentage of neutrophil migrating directionally in response to C5a gradients is maximal at 0.1 to 10 µM C5a in the end chamber. Neutrophil chemotaxis increased with increasing C5a concentration from 1 nM to 1 µM. 1 nM, 10 nM, 50 nM, 100 nM, 1 µM and 10 µM (N=3, N=3, N=3, N=6, N=3, N=3, respectively) (*p < 0.05, ***p < 0.001; Kruskal-Wallis with Dunn’s test). [file Presentation_1.pdf]

Supplementary Information

Human neutrophils respond to complement activation and inhibition in microfluidic devices

Sinan Muldur[1,2], Douangsone D. Vadysirisack[3], Sharan Ragunathan[3], Yalan Tang[3], Alonso Ricardo[3], Camil E. Sayegh[3], and Daniel Irimia[1,2]*

[1] BioMEMS Resource Center, Department of Surgery, Massachusetts General Hospital, Harvard Medical School, Boston, MA 02129, USA

[2] Shriners Burns Hospital, Boston, MA 02114, USA

[3] Ra Pharmaceuticals, Inc., Cambridge, MA 02140, USA (now a part of UCB Pharma)

* Please address correspondence to dirimia@mgh.harvard.edu

Supplementary figures:

Figure S1. Percentage of neutrophil migrating directionally in response to C5a gradients is maximal at 0.1 to 10 μM C5a in the end chamber.

Figure S2. Effect of serum on C5a-induced neutrophil chemotaxis.

Figure S3. Effect of ECU, AVA, and RA101295 on neutrophil recruitment and *S. aureus* particle phagocytosis when the inhibitors are present everywhere in the device versus inside the central reservoirs only.

Figure S4. Kinetics of neutrophil recruitment and phagocytosis of *S. aureus* -particles in the presence of ECU, AVA, and RA101295.

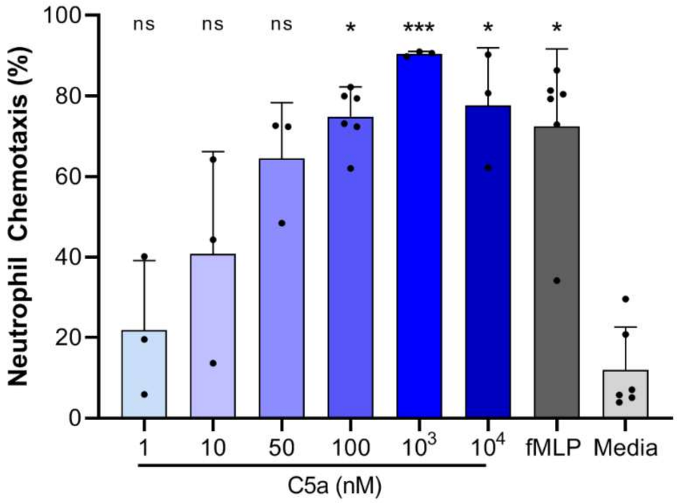

Figure S1. Percentage of neutrophil migrating directionally in response to C5a gradients is maximal at 0.1 to 10 µM C5a in the end chamber. Neutrophil chemotaxis increased with increasing C5a concentration from 1 nM to 1 µM. 1 nM, 10 nM, 50 nM, 100 nM, 1 µM and 10 µM (N=3, N=3, N=3, N=6, N=3, N=3, respectively) (*$p < 0.05$, ***$p < 0.001$; Kruskal-Wallis with Dunn's test).

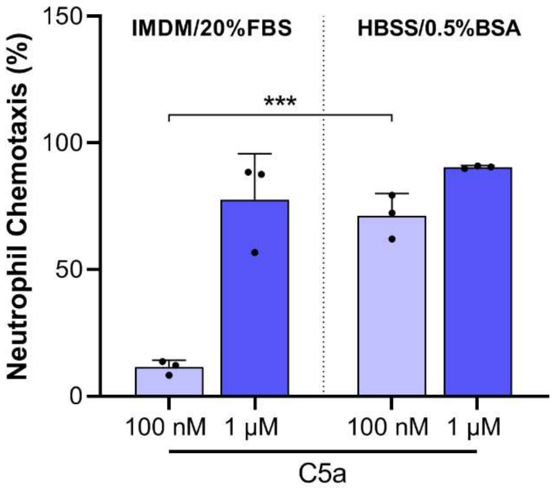

Figure S2. Effect of serum on C5a-induced neutrophil chemotaxis. Fewer neutrophils respond to C5a at 100 nM in IMDM with 20% FBS compared to serum-free HBSS with 0.5%BSA (***$p < 0.001$; unpaired two-tailed *t*-test). This effect was not observed for higher C5a doses (1 μM, N=3).

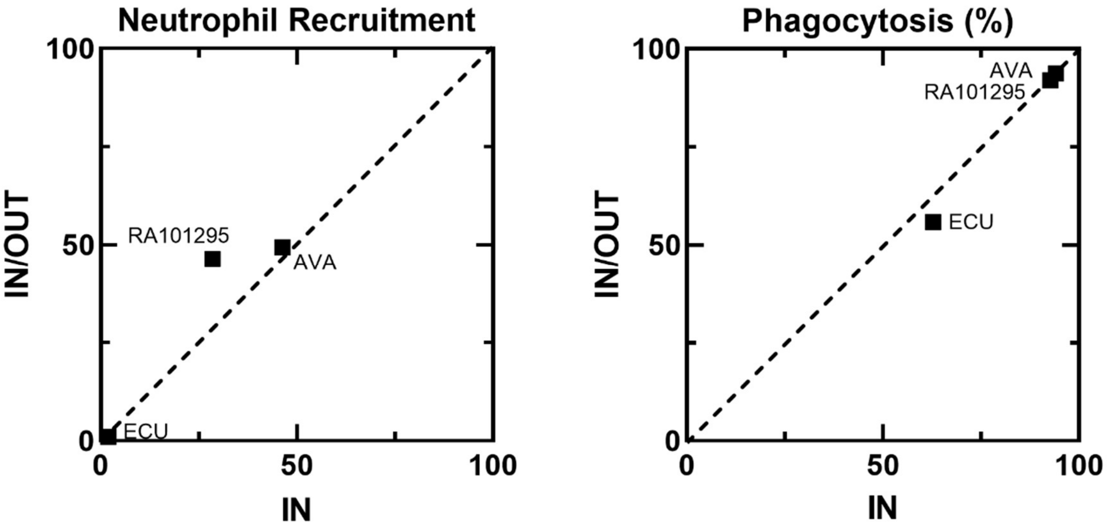

Figure S3. Effect of ECU, AVA, and RA101295 on neutrophil recruitment and *S. aureus* particle phagocytosis when the inhibitors are present everywhere in the device versus inside the central reservoirs only. We observed the same effect when ECU and AVA were inside the chambers only (with plasma) or when present everywhere in the device (with neutrophils). RA101295, when present everywhere and with cells, slightly increased neutrophil recruitment. **(N=3)**

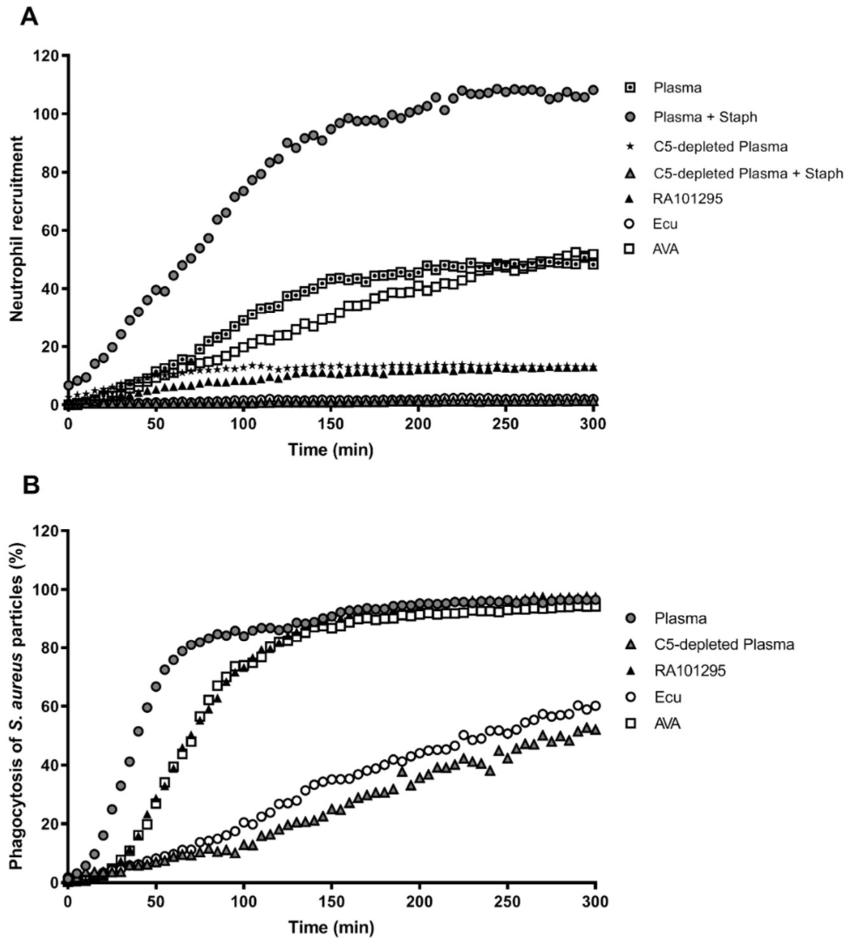

Figure S4. Kinetics of neutrophil recruitment and phagocytosis of *S. aureus* -particles in the presence of ECU, AVA, and RA101295. A. Neutrophil recruitment over time. The fastest and strongest neutrophil recruitment was obtained in the positive control (Plasma + *S. aureus*), while the lowest recruitment was obtained in the negative control (C5-depleted + *S. aureus*) and ECU pretreated plasma. RA101295 and AVA reduced neutrophil recruitment as well but to a lesser extent. **B.** Phagocytosis of *S. aureus* particles by the recruited neutrophils. The fastest phagocytosis of *S. aureus* particles was observed in the positive control (Plasma). Phagocytosis was delayed in the AVA and RA10295 conditions, even though they ultimately reached the same phagocytosis percentages as the positive control. Neutrophils in ECU-pretreated plasma followed a much slower and diminished phagocytosis rate similar to the negative control (C5-depleted) (N = 1).
